# The Role of the Rare Variants in the Genes Encoding the Alpha-Ketoglutarate Dehydrogenase in Alzheimer’s Disease

**DOI:** 10.3390/life11040321

**Published:** 2021-04-06

**Authors:** Dora Csaban, Klara Pentelenyi, Renata Toth-Bencsik, Anett Illes, Zoltan Grosz, Andras Gezsi, Maria Judit Molnar

**Affiliations:** 1 Institute of Genomic Medicine and Rare Disorders, Semmelweis University, H-1082 Budapest, Hungary; csaban.dora@med.semmelweis-univ.hu (D.C.); grosz.zoltan@med.semmelweis-univ.hu (Z.G.); 2 PentaCore Laboratory Budapest, H-1094 Budapest, Hungary; 3 Department of Measurement and Information Systems, Budapest University of Technology and Economics, H-1117 Budapest, Hungary

**Keywords:** alpha-ketoglutarate dehydrogenase complex, αKGDHc, Alzheimer, dementia, DLD, rare variants, brain tissue

## Abstract

There is increasing evidence that several mitochondrial abnormalities are present in the brains of patients with Alzheimer’s disease (AD). Decreased alpha-ketoglutarate dehydrogenase complex (αKGDHc) activity was identified in some patients with AD. The αKGDHc is a key enzyme in the Krebs cycle. This enzyme is very sensitive to the harmful effect of reactive oxygen species, which gives them a critical role in the Alzheimer and mitochondrial disease research area. Previously, several genetic risk factors were described in association with AD. Our aim was to analyze the associations of rare damaging variants in the genes encoding αKGDHc subunits and AD. The three genes (*OGDH*, *DLST*, *DLD*) encoding αKGDHc subunits were sequenced from different brain regions of 11 patients with histologically confirmed AD and the blood of further 35 AD patients. As a control group, we screened 134 persons with whole-exome sequencing. In all subunits, a one–one rare variant was identified with unknown significance based on American College of Medical Genetics and Genomics (ACMG) classification. Based on the literature research and our experience, R263H mutation in the *DLD* gene seems likely to be pathogenic. In the different cerebral areas, the αKGDHc mutational profile was the same, indicating the presence of germline variants. We hypothesize that the heterozygous missense R263H in the *DLD* gene may have a role in AD as a mild genetic risk factor.

## 1. Introduction

Alzheimer’s disease (AD), the most frequent primary dementia (60–80%), is a neurodegenerative disease associated with initial memory impairment and cognitive deterioration, which may affect later visuospatial orientation, behavior, and speech as well [1,2]. AD is usually a complex multifactorial disease, although monogenic forms are known, too. In the background of multifactorial AD, in addition to genetic risk factors, many pathophysiological dysfunctions are described such as oxidative stress, problems with the cell cycle, and neurovascular dysfunction [3].

A significant proportion of AD cases (90–95%) are sporadic and belong to the late-onset Alzheimer’s disease (LOAD) group (>65 years). Several genetic risk factors have already been identified through genome-wide association studies (GWAS) and whole-exome sequencing (WES) in association with LOAD. The most significant of these is the apolipoprotein E (*APOE*) 4 allele. In addition, several genetic risk genes in LOAD, such as *TREM2* and *ADAM10*, have been shown to not only directly affect tau and amyloid precursor protein (*APP*) but also modulate endocytosis, immune response, and cholesterol metabolism [1]. Early-onset Alzheimer’s disease (EOAD) is often of monogenic origin, and the symptoms of the patients are already present in the 40s (in extreme cases the 30s); about 10% of all AD patients belong here. While the LOAD, which has a complex, heterogeneous etiology has 70–80% inheritance, the EOAD, which is usually a genetically deterministic form, has 92–100% inheritance. However, only 5–10% of EOAD cases are explained by high-penetrant mutations in the three known EOAD genes: *APP*, presenilin 1 and 2 (*PSEN1, PSEN2*). However, the majority of AD cases are still unresolved, indicating the need to identify additional causal or risk genes. Understanding the role of various known and newly described risk genes has greatly contributed to the understanding of the pathomechanism of AD and shed light on the molecular pathways involved [4,5,6,7].

The accumulation of aggregated extracellular amyloid-β (Aβ) peptides and intracellular neurofibrillary tangles (NFTs) made of abnormally hyperphosphorylated tau are the fundamental hallmarks of AD neuropathology [8]. The tau lesion is correlated with cognitive disturbances, suggesting a fundamental role of tau pathology in neurodegeneration of this disease [9].

There is an increasing evidence that several mitochondrial abnormalities are present also in the brain of patients with AD [10,11]. Impaired energy metabolism, disrupted mitochondrial bioenergetics, abnormal balance of mitochondrial fission and fusion, axonal trafficking, and mitochondrial distribution have been described in association with AD [12]. Furthermore, there are observations that are supporting the impaired mitochondrial biogenesis, endoplasmic reticulum–mitochondrial interaction, mitophagy, and mitochondrial proteostasis also in AD [12]. A recent publication associates the mitochondrial dysfunction with tau pathology in AD [13]. The axonal transport of different organelles and mitochondrial dynamics can be damaged by the overexpression of hyperphosphorylated and aggregated tau, resulting in mitochondrial dysfunction [14].

Positron emission tomography with 2-(^18^F) fluoro-2-deoxy-d-glucose (FDG PET) investigations have been detected glucose hypometabolism in the early phase of AD in the brain [15]. This was interpreted as impaired energy metabolism through oxidative phosphorylation. The decreased glucose metabolism correlated with the reduced levels of blood thiamine diphosphate, which is a critical coenzyme of pyruvate dehydrogenase complex (PDHc), as well as α-ketoglutarate dehydrogenase complex (αKGDHc) in the Krebs cycle and transketolase in the pentose phosphate pathway in the frontal, temporal, and parietal cortical regions of the patients with AD [16]. In patients with primary mitochondrial disease, the FDG PET detected impaired cerebral glucose uptake in the temporal and occipital lobe [17]. Inczedy et al. reported in their case series that the systematic evaluation of patients with mtDNA mutations evidenced cognitive deficits quite similar to those commonly seen in AD [18].

In this study, we aimed to investigate the role of αKGDHc in the pathogenesis of AD. This enzyme is a key player in the Krebs cycle, and alpha-ketoglutarate catalyzes an irreversible reaction converting alpha-ketoglutarate, coenzyme A and NAD+ to succinyl-coenzyme A, NADH and CO_2_, using thiamine pyrophosphate as a cofactor. This enzyme is located in the mitochondrial matrix and uses thiamine pyrophosphate as a cofactor. The enzyme consists of three subunits encoded by the *OGDH* (E1 subunit: 2-oxoglutarate dehydrogenase), *DLST* (E2 subunit: dihydrolipoamide succinyltransferase), and *DLD* (E3 subunit: dihydrolipoamide dehydrogenase: LADH) genes. Previous investigations into the cell-specific localization of subunits in the adult human brain cortex have also revealed that *DLD* was identified in both neurons and glia, while *OGDH* and *DLST* were detected only in neurons [19]. The impairment of LADH function affects numerous key metabolic routes, as it is a common E3 subunit to the alpha-ketoglutarate, alpha-ketoadipate, pyruvate, and branched-chain alpha-keto acid dehydrogenase complexes. This is also due to the clinical severity of loss in LADH function [20]. Previous in vitro experiments have demonstrated that disease-causing variants show increased reactive oxygen species (ROS)-generating capacities as compared to wild-type enzyme [21]. Based on the first high-resolution structural study of the E3 subunit mutant, it is hypothesized, for example, that mutations in the dimeric interface are likely to modify the geometry and polarity of E3; thus, they may lead to enhanced ROS generation [22]. Other studies of E3 have also revealed that the variant with the most severe structural aberrations (P453L-E3) has the most serious clinical symptoms, while the variant with the least abnormity (G426E-E3, G194C-E3) has a mild clinical manifestation [23].

In this study, we focused on αKGDHc, which is a key enzyme in the Krebs cycle that is very sensitive to the harmful effect of ROS, which gives them a critical role in Alzheimer’s and mitochondrial disease research areas. It has previously been demonstrated that αKGDHc activity decreases in AD [24,25]. In the brain, it behaves differently compared to other tissues—it is implicated in glutamate degradation, having an important role in neurotoxicity [26]. The enzyme activity is also different in each brain region, the highest of which is in the cortex [27]. Cholinergic neurons are rich in αKGDHc complexes, and they are extremely sensitive to αKGDHc defects [26]. Some research suggests a correlation between lower αKGDHc activity and more severe dementia (CDR value: clinical dementia rating) [28].

E3-deficiency, inherited in autosomal recessive form, is a severe infantile lethal disease. The E3 defect affects mostly tissues with high oxygen expenditure, resulting in neurological and cardiological symptoms. Typical symptoms are hypotonia, developmental delay, encephalopathy, recurrent metabolic crises, and lactic acidosis. The symptoms usually appear at a young age with lactate acidosis and hypotonia. The affected children usually die under 4–10 years of age due to their first or recurrent metabolic decompensation [29,30,31].

Alpha-KGDH defects have been observed in neurodegenerative diseases as Alzheimer’s, Parkinson’s, and SCA1 [32]. We suppose that the heterozygous mutations of αKGDHc’s subunits could be genetic risk factors or triggers of AD.

Waqar et al. investigated the association between AD and *DLD* through tau-mediated toxicity in a C. elegans model of AD. Based on their results, *DLD* suppression leads to significant tau phosphorylation, thereby influencing the pathology of AD [33].

## 2. Materials and Methods

### 2.1. Patients

**Figure 1:**
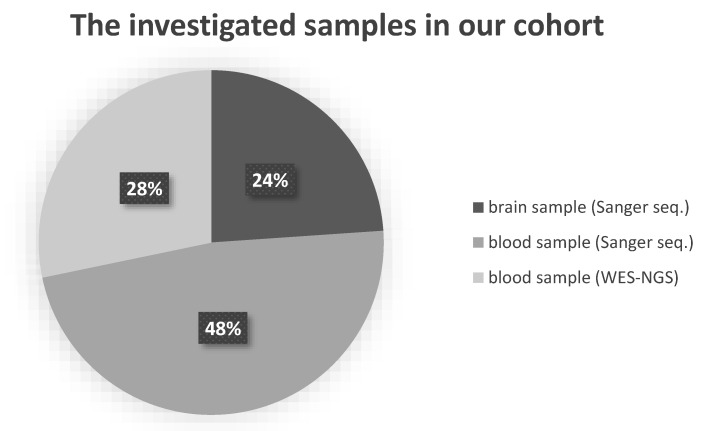
The distribution of the different analyzed samples by sample type. Abbreviations: WES-NGS: whole-exome sequencing with next-generation sequencing.

For 46 patients diagnosed with AD, all three subunits of αKGDHc were genetically analyzed. In 11 patients diagnosed with AD, post-mortem brain tissue was examined (5 male, 69.5 ± 9 years; 6 female, 77 ± 8 years). Brain samples were selected from the Human Brain Tissue Bank of Semmelweis University (HBTB); in all cases, detailed neuropathological investigation certified the Alzheimer’s diagnosis. HBTB has been authorized by the Committee of Science and Research Ethics of the Hungarian Ministry of Health (No. 6008/8/2002/ETT) and the Semmelweis University Regional Committee of Science and Research Ethics (No. 32/1992/TUKEB). For autopsy, brains were removed from the skull with a post-mortem delay of 2–6 h. Several regions (frontal, temporal Brodman 20–21, prefrontal Brodman 9, parietal, and parahippocampal lobes) of brain tissues were analyzed per sample to detect rare damaging variants and to observe possible somatic mutations. Further AD patients were selected from our NEPSYBANK [34]. These patients were diagnosed with AD by a board-certified neurologist. On blood samples of 13 AD patients (2 male, 72 ± 13 years; 11 female, 62 ± 8 years), whole-exome sequencing with next-generation sequencing (WES-NGS) was performed, and 22 AD patients (6 male, 63 ± 4.6 years; 16 female, 55.8 ± 10 years) were analyzed with bidirectional Sanger sequencing. The distribution of the analyzed samples between the different sample types and sequencing methodologies is shown in Figure 1. As a control group, we investigated brain tissues from patients without any sign of neurodegenerative disorders from the HBTB. This healthy control group consisted of 9 post-mortem brain tissues (3 male, 73 ± 5 years; 6 female, 54 ± 14 years) in which histopathological examinations did not detect any alterations indicating neurodegeneration. The 134 control individuals (72 male; 62 female) could be divided into two groups: 55 were healthy, in 79 cases, no neurological disorders were detected. They were investigated by WES-NGS.

### 2.2. Genetic Investigations

All DNA from frozen brain samples in our study was isolated with a QIAamp DNA tissue kit according to the manufacturer’s instructions (QIAgen, Hilden, Germany). DNA from blood samples were isolated with a QIAamp DNA blood kit according to the manufacturer’s instructions (QIAgen, Hilden, Germany). We analyzed the third subunit of αKGDHc with bidirectional Sanger sequencing using an ABI Prism 3500 DNA Sequencer (Applied Biosystems, Foster City, CA, USA) at 22 AD patients. The sequences were compared with the human reference genome using NCBI’s Blast^®^ application (*OGDH*: (GRCh37/hg19, ENST00000222673.5, NM_002541.4, *DLST*: GRCh37/hg19, ENST00000334220.4, NM_001933.5, *DLD*: GRCh37/hg19, ENST00000205402.5, NM_000108.5). DNA library preparation for WES was performed by a SureSelect QXT library preparation kit (Agilent Technologies, Santa Clara, CA, United States) according to the manufacturer’s instructions. After library preparation, next-generation sequencing (NGS) was performed on the Illumina HiSeq 2500 system (Illumina, San Diego, CA, USA). First, cluster generation was performed on the cBot using HiSeq PE (Paired-End) Cluster Kit v4, then for sequencing HiSeq SBS Kit Reagent v4 was used.

### 2.3. Bioinformatic Analysis

After qualitative filtering of the raw data, the sequences were aligned with the GRCh37/hg19 reference genome using the BWA-MEM (Burrows-Wheeler Aligner; version 0.7.15) default parameters [35]. Variant calling from the NGS data was carried out with GATK HaplotypeCaller (Genome Analysis Toolkit, version 3.3-0) following the GATK Best Practices Guidelines [36]. Variant Call Format (VCF) files were annotated with different types of annotations by VariantAnalyzer software developed by the Budapest University of Technology and Economics. The annotations of SNPs and short INDELs were performed using for example SnpEff [37], which predicted their effect on genes and using ClinVar for disease associations [38]. Filtration for potentially damaging variants was performed VariantAnalyzer software. Variants with minor allele frequencies (MAFs) not exceeding 5% and less than 5% in our in-house exome database were also considered. The MAF was determined based on data of the 1000 Genomes Project (1 KG), the Genome Aggregation Database (GnomAD v2.1). During the next step of the analysis, rare alterations were filtered for known disease-causing alterations and then for non-synonymous variants. Variants selected based on these criteria were analyzed in our control group and reserved for further analysis. Interpretation of the novel variants were prepared according to the American College of Medical Genetics and Genomics (ACMG) guideline [39,40]. Variants were classified using the Varsome and Franklin websites [41,42].

## 3. Results

Analyzing the subunits of the enzyme for 46 patients with AD, we found three probably damaging missense mutations—in all subunits, one missense mutation was present. In this AD group, *OGDH* harbored eight synonymous rare variants (E115E; S132S; L228L; H425H; T434T; S603S; T996T; N1021N), two benign or likely benign rare missense variants (S55L; V1018I), and one rare variant with unknown significance (P471H). In the *DLST* gene, only one variant with unknown significance was detected (P204L). Only one rare missense substitution was detected in the *DLD*. All alterations found in our AD cohort are presented in Table 1.

In the control group, altogether, 29 exonic rare variants were found in the *OGDH* gene (synonymous n = 23, missense n = 6), 2 in the *DLST* gene (missense n = 2), and 10 in the *DLD* gene (synonymous n = 9, missense n = 1). These missense alterations are all benign or likely benign variants according to ACMG, except for the *DLST* I393V. The *DLST* I393V alteration is classified as VUS (variant of uncertain significance) by ACMG, because it is not found in GnomAD. The rare alteration was identified in a healthy 70-year-old male.

The characteristics of the detected rare variants and VUS in the AD group are the following.

In the *DLD* gene (αKGDHc 3. subunit), exon 9 c.788 G > A (R263H, rs145670503) mutation was detected in the frontal, parahippocampal, and temporal lobes of one patient, who died at age 64. The diagnosis of Alzheimer’s in this patient was proven with histopathological analysis. At codon 263 of the DLD protein, arginine was replaced with histidine. This variant is present with low allele frequency in population databases (GnomAD 0.0008, ExAC 0.0007, 1000G 0.0004). According to the predictions software, the variant is pathogenic. Based on the available evidence, ACMG classifies it as a variant of uncertain significance, but previously, this alteration was detected in a CLA (Congenital Lactic Acidosis) patient and interpreted as a disease-causing mutation in a biallelic form [30]. In the healthy control screening, we did not find this mutation.

In the *OGDH* gene, only one rare missense variant was detected, which is classified as VUS by ACMG. The P471H alteration is not included in the population databases and was present neither in our control group nor in the literature. Prediction software qualified it as pathogenic; however, most of the clinically reported missense variants in the *OGDH* gene are known as benign (37 out of 44, 84.1%).

In the *DLST* gene (αKGDHc 2. subunit) in exon 9, we detected the c. 611 C > T (P204L, rs142872233) missense mutation, causing a proline/leucine amino acid change (P204L). Based on most prediction software, the alteration was classified as damaging and low MAF value (GnomAD 0.017, ExAC 0.016, 1000G 0.008). ‘MutationTaster’ and ‘SIFT’s predictions are disease-causing; it is a conserved amino acid. The allele frequency is low but higher than 0.001 (GnomAD 0.0017). Based on these, ACMG classifies the variant as uncertain significance. In the course of healthy control’s testing (n = 24), we found this mutation in a frozen brain sample with a lack of any neuropathological lesions typical for dementia (this patient died at age 74 in acute circulatory failure). It was present in an additional three individuals in our WES control group.

No differences were found regarding the presence or lack of mutations in the different analyzed brain regions.

## 4. Discussion

The αKGDHc mutation testing detected a heterozygous missense mutation (R263H) in the *DLD* gene, which was supposed to be damaging based on the lack of large genomic healthy control investigations. The significance of this variant is supported by the observation that the deficiency of αKGDHc and PDHc enzymes in the Krebs cycle may explain the glucose metabolism defect observed in the brains of AD patients [43]. Furthermore, impaired mitochondrial function has been described as an early and outstanding feature of the AD, suggesting that mitochondria play a fundamental role in the pathogenesis of disease [12]. Due to mitochondrial dysfunction, tau is phosphorylated and aggregated, while hyperphosphorylated tau damages mitochondrial axonal transport, creating a vicious cycle that impairs nerve and synaptic functions, leading to memory impairment in AD [13].

The R263H alteration was identified in heterozygous form in all investigated regions of our patient’s brain, excluding the presence of a somatic mutation. This alteration is localized at a conserved nucleotide position; many types of prediction software score a pathogenic amino acid change (SIFT, PolyPhen2, Mutation Taster). This alteration was published in biallelic form in a severe patient’s primary mitochondrial disease, whose main symptoms were psychomotor retardation and epileptic encephalopathy [30]. This was the only reference to this variant pointing out its rarity. The difference between the two clinical manifestations may be explained by the presence of mono/biallelic form. Interestingly, the same mutation was found in one of our patients with suspected primary mitochondrial disease heterozygous form (unpublished data). This patient had early onset epilepsy, spastic paraparesis, and cognitive dysfunction. More brain samples should be investigated for major conclusions to support our risk factor theory.

Several substitutions were reported as VUS in the vicinity of the variant detected by us (c.788G > A, R263H) in the *DLD* gene. These are c.781T > G (F261V), c.782T > G (F261C), c.783T > G (F261L), and c.787C > A (R263S). This locus is associated with dihydrolipoamide dehydrogenase deficiency and is a TF-binding site for PRDM4 (involved in cell differentiation). The GnomAD’s frequency data show a higher occurrence of R263H but no homozygous appearance in the European population [44].

So far in the *DLD* gene, 14 disease-causing substitutions are known in the literature: (I47T, K72E, G229C, G252C, G328C, M361V, E375K, I393T, E398K, I441T, D479V, R482K or R482G, P488L, and R495G) [20]. Based on X-ray crystallographic analysis, the described pathogenic mutations are localizing at the cofactor-binding site, or disulfide-exchange site, or on the homodimer interface domain [45]. The R263H amino acid change is localizing at *DLD*’s NAD^+^/NADH-binding region; in this region, only one mutation is described: G194C amino acid change. According to the literature, most mutations are localizing in the *DLD* interface region. Mutations in the interface domain (I480M) or NAD^+^/NADH-binding domain (G229C) cause milder symptoms [46]. Odièvre et al. reported similar mutations in the interface domain (R482G, D479V) with E3 deficiency without clinical and biochemical evidence [47].

Inhibition of dihydrolipoamide dehydrogenase (DLD) results in increased phosphorylation of tau in nematode C. elegans—a model of AD [33]. A linkage association exists between AD and the chromosomal region of the *DLD* gene [48].

Mastrogiacomo et al. measured the activity of αKGDHc complex at post mortem AD brain samples. Compared with the controls, the activity was reduced at different levels in the different lobes [49].

However, the heterozygous rare variants of the *OGDH* and *DLST* are classified as variants with unknown significance by ACMG. We suppose that the *DLST* P204L alteration may be benign, since it is present in several control persons. Furthermore, for the rare variant in the *OGDH* gene (P471H), we found no evidence that it could play a role as a risk factor in the development of AD.

We did not find any genetic differences between various lobes indicating the presence of germline mutation.

We conclude that the rare variants of the heterozygous αKGDHc subunits can be rare genetic risk factors for AD enhancing the accumulation of tau. Since in the difference cerebral areas, the mutation profile of the αKGDHc was the same, we do not assume that somatic mutations are responsible for neuropathological alterations.

## Figures and Tables

**Table 1:** The detected variants in the investigated cohort.

Gene	Variant ID	Variant Effect	Clinical Significance	ACMG Classification	MAF (GnomAD, Non-Finnish)	Patients	Controls	Ref
OGDH	c.164 C > T p. S55L	missense	B	Likely Benign	1.07%	2/46	4/134	-
	c.345 A > G E115E	synonymous	B	Benign	2.47%	1/46	18/134	-
	c.396 G > A S132S	synonymous	B	Benign	4.88%	1/46	5/134	-
	c.682 C > T L228L	synonymous	B	Likely Benign	-	2/46	0/134	-
	c.1275 C > T H425H	synonymous	B	Likely Benign	-	1/46	0/134	-
	c.1302 T > A T434T	synonymous	B	Likely Benign	-	1/46	0/134	-
	c.1412 C > A P471H	missense	B	Uncertain Significance	-	1/46	0/134	-
	c.1809 C > A S603S	synonymous	B	Likely Benign	-	1/46	0/134	-
	c.2988 C > T T996T	synonymous	B	Benign	5.04%	1/46	10/134	-
	c.3052 G > A V1018I	missense	B	Benign	5.05%	2/46	12/134	-
	c.3063 C > T N1021N	synonymous	B	Benign	5.05%	1/46	10/134	-
DLST	c.611 C > T P204L	missense	B	Uncertain Significance	2.27%	1/46	4/134	-
DLD	c.788 G > A R263H	missense	D	Uncertain Significance	≤0.01	1/46	0/134	[30]

Abbreviations: OGDH: oxoglutarate dehydrogenase; DLST: dihydrolipoamide S-succinyltransferase; DLD: dihydrolipoamide dehydrogenase; ACMG: American College of Medical Genetics and Genomics; GnomAD: Genome Aggregation Database; B: Benign; D: Damaging.

## Data Availability

Data supporting reported results (datasets analyzed or generated during the study) can be found at the data warehouse of the Institute of Genomic Medicine and rare Disorders.
